# New Power MOSFET with Beyond-1D-Limit *R*_SP_-*BV* Trade-Off and Superior Reverse Recovery Characteristics

**DOI:** 10.3390/ma13112581

**Published:** 2020-06-05

**Authors:** Meng Zhang, Baikui Li, Jin Wei

**Affiliations:** 1 Key Laboratory of Optoelectronic Devices and Systems of Ministry of Education and Guangdong Province, College of Physics and Optoelectronic Engineering, Shenzhen University, Shenzhen 518060, China; sara.zhang@connect.polyu.hk; 2 Institute of Microelectronics, Peking University, Beijing 100871, China; jin.wei@pku.edu.cn

**Keywords:** power MOSFET, deep trench, p-islands, beyond-1D-limit, reverse recovery

## Abstract

The application of conventional power metal-oxide-semiconductor field-effect transistor (MOSFET) is limited by the famous one-dimensional “silicon limit” (1D-limit) in the trade-off relationship between specific on-resistance (*R*_SP_) and breakdown voltage (*BV*). In this paper, a new power MOSFET architecture is proposed to achieve a beyond-1D-limit *R*_SP_-*BV* trade-off. Numerical TCAD (technology computer-aided design) simulations were carried out to comparatively study the proposed MOSFET, the conventional power MOSFET, and the superjunction MOSFET. All the devices were designed with the same breakdown voltage of ~550 V. The proposed MOSFET features a deep trench between neighboring p-bodies and multiple p-islands located at the sidewall and bottom of the trench. The proposed MOSFET allows a high doping concentration in the drift region, which significantly reduces its *R*_SP_ compared to the conventional power MOSFET. The multiple p-islands split the electric field into multiple peaks and help the proposed MOSFET maintain a similar breakdown voltage to the conventional power MOSFET with the same drift region thickness. Another famous device technology, the superjunction MOSFET (SJ-MOSFET), also breaks the 1D-limit. However, the SJ-MOSFET suffers a snappy reverse recovery performance, which is a notorious drawback of SJ-MOSFET and limits the range of its application. On the contrary, the proposed MOSFET presents a superior reverse recovery performance and can be used in various power switching applications where hard commutation is required.

## 1. Introduction

The power metal-oxide-semiconductor field-effect transistor (MOSFET) is a commonly used power switch in power switching applications. To obtain a higher breakdown voltage (*BV*) in the conventional power MOSFET, the drift region has to be thicker and more lightly doped, which leads to a higher specific on-resistance *R*_SP_ (= *R*_DS-ON_ × area). Thus, there is a trade-off relationship between *R*_SP_ and *BV*, which is often referred to as the “silicon limit” or “1D-limit”: *R*_SP_ ∝ *BV*^2.5^ [1,2,3]. As a consequence, when the blocking voltage of the power MOSFET increases, its *R*_SP_ rises quickly. For blocking voltages above 200~300 V, conventional power MOSFETs are not preferred due to the high *R*_SP_.

The superjunction MOSFET (SJ-MOSFET) features laterally repeated p/n pillars, which assists the voltage blocking capability by lateral depletion [4,5,6,7]. Thus, the doping concentration of the pillars can be much higher than that of the drift region in conventional power MOSFET. In off-state, the electric field in the SJ-MOSFET keeps almost constant along the depth of the n/p pillars, so the pillars can be much shorter than the drift region of the conventional power MOSFET for the same *BV*. Therefore, the SJ-MOSFET breaks the 1D-limit in *R*_SP_-*BV* trade-off, owing to the shorter and more heavily doped n-pillar compared to the drift region of conventional power MOSFET [8,9,10,11]. The first SJ-MOSFET was demonstrated in 1998 [9]. A key to the development of SJ-MOSFET is the scaling of the p/n-pillars, which has been steadily advancing with the progress of fabrication technology. After around twenty years’ development, the most advanced SJ-MOSFET boasts a *R*_SP_ as low as 10 mΩ·cm^2^ for a 600-V voltage rating, which is over five times lower than the theoretical *R*_SP_ according to the silicon limit [4,12]. However, the large aspect PN junction area of the SJ-MOSFET leads to snappy reverse recovery performance and prohibits its adoption in many applications [13,14,15]. Although various approaches have been proposed to improve the performance of body diode in conventional power MOSFETs and SJ-MOSFETs, such as carrier lifetime control [16,17], integration of Schottky diodes [18,19,20], usage of anti-paralleled freewheeling diode [21,22,23], and injection control using channel diode [24,25], the snappy reverse recovery of the SJ-MOSFET remains an unsolved issue since it is intrinsic to the SJ-MOSFET structure.

In this paper, a new power MOSFET is proposed to obtain a beyond-1D-limit *R*_SP_-*BV* trade-off and a superior reverse recovery performance. The proposed MOSFET features a deep trench and multiple p-islands located at the trench sidewall. The proposed MOSFET was comprehensively studied using numerical TCAD (technology computer-aided design) device simulations and mixed-mode circuit simulations and compared with a conventional power MOSFET and a SJ-MOSFET. All the MOSFETs were designed with a breakdown voltage of ~550 V. The proposed MOSFET allows a higher doping concentration in the drift region, which significantly reduces the *R*_SP_. The multiple p-islands split the electric field peak of the conventional power MOSFET into multiple peaks so that a high breakdown voltage is maintained. A beyond-1D-limit *R*_SP_-*BV* trade-off was achieved in the proposed MOSFET. Furthermore, the notorious snappy reverse recovery performance of a SJ-MOSFET is avoided in the proposed MOSFET, which allows the proposed MOSFET to be adopted in a much wider range of applications.

The simulations are based on Sentaurus TCAD [26]. The Sentaurus Structure Editor is used for structure and mesh construction. The Sentaurus Device is used for device simulations and mixed-mode circuit simulations. The mesh of the structures contains four parts: an overall mesh for the whole device structure with lateral/vertical mesh sizes of 0.4/0.5 µm; a refined mesh with much smaller lateral/vertical mesh sizes (0.1/0.2 µm) is placed at the top 2-µm region; a further refined mesh with a size of 0.01 µm is placed around the PN junctions; the oxide/silicon interface is refined with a mesh size of 0.002 µm along the direction perpendicular to the interface. In the Sentaurus Device simulations, the electron/hole continuity equations and Poisson equations are solved self-consistently. The mixed-mode circuit simulations are carried out with the circuit equations solved in the same manner as traditional SPICE tools. Shockley-Reed-Hall and Auger combination, impact ionization (Okuto model), doping-dependent transport, high-field saturation effects, band narrowing, are all considered.

## 2. Device Structure and *R*_SP_-*BV* Trade-Off

**Figure 1:**
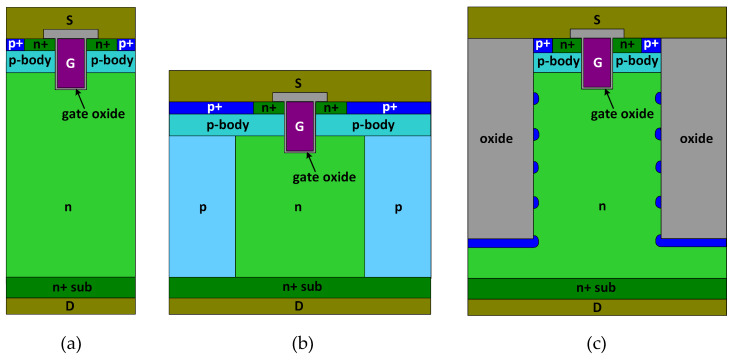
Schematic cross-sectional structures of (**a**) conventional power MOSFET (metal-oxide-semiconductor field-effect transistors), (**b**) SJ-MOSFET, and (**c**) proposed MOSFET featuring deep trench and p-islands.

Figure 1 plots the schematic cross-sectional structures of the conventional power MOSFET, the SJ-MOSFET, and the proposed MOSFET. The proposed MOSFET has a deep trench between neighboring p-bodies. Multiple p-islands are located at the sidewall and bottom of the trenches. The number of p-islands was designed according to the requirement of *BV*. In this study, 5 p-islands were adopted. The doping concentration of the p-islands is 2.5 × 10^18^ cm^−3^. The distance between adjacent p-islands is 6.6 µm. The deep trench is filled with an insulator (silicon oxide is used in this paper). All the studied MOSFETs include a 1-µm-wide gate in one cell pitch. The gate oxide thickness is 80 nm. The channel length is 0.7 µm. The n/p-pillars in SJ-MOSFET have a doping concentration of 4 × 10^15^ cm^−^^3^, and a thickness of 42 µm. The key device parameters of the studied MOSFETs are listed in Table 1.

**Figure 2:**
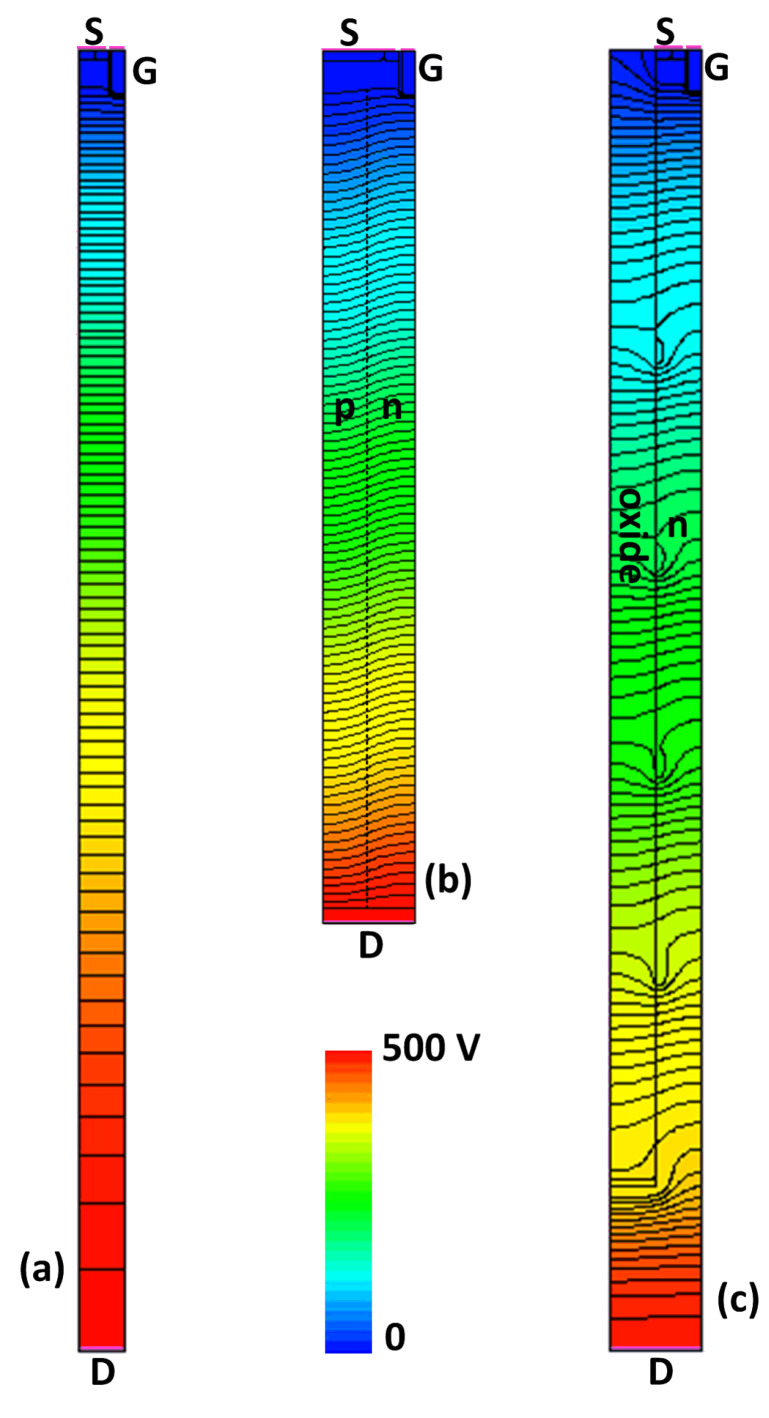
Electrostatic potential contour under *V*_DS_ = 500 V in off-state: (**a**) conventional MOSFET, (**b**) SJ-MOSFET, and (**c**) the proposed MOSFET.

**Figure 3:**
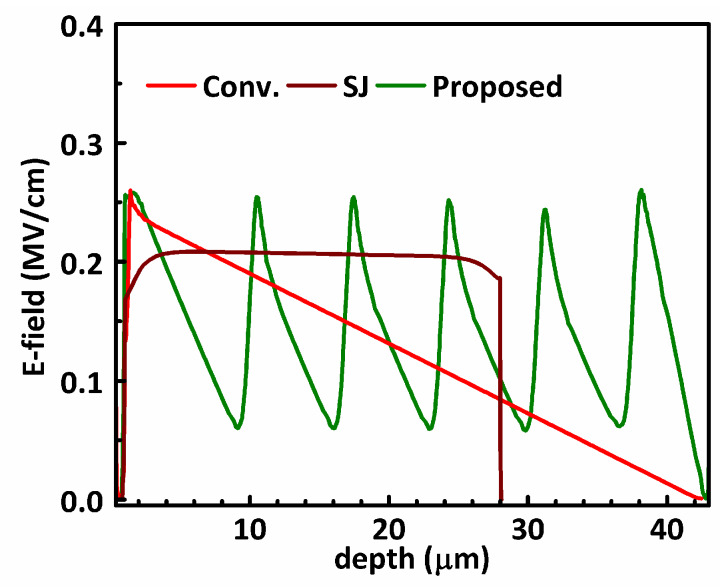
Electric field along the depth of the studied MOSFETs at *V*_DS_ = 500 V in off-state.

To understand the mechanism for the proposed MOSFET to break the 1D-limit, the two-dimensional electrostatic potential contours of the studied MOSFETs are plotted in Figure 2, and the electric field along the depth of the devices are plotted in Figure 3. The devices are in off-state under *V*_DS_ = 500 V. For the conventional power MOSFET, the electrostatic potential lines are crowded around p-body/n-drift junction, and the distance between adjacent equal-potential lines is gradually increasing towards the lower side of the drift region. From Figure 3, the electric field in the conventional power MOSFET has a triangle shape, peaking near the top of the device. When the electric field peak reaches the critical breakdown field, the breakdown voltage is the integration of the triangle-shaped electric field distribution along the depth.

For the SJ-MOSFET, the p/n pillars are fully depleted laterally with a low off-state voltage, even though the doping concentration in the p/n pillars of SJ-MOSFET are much higher than conventional power MOSFET. At higher off-state voltage, the fully depleted p/n pillars behave like dielectrics. From Figure 2, the equal-potential lines in the SJ-MOSFET at *V*_DS_ = 500 V are more evenly distributed along the depth compared to conventional power MOSFET. The electric field in the SJ-MOSFET has a rectangle shape; thus, for the same *BV*, the SJ-MOSFET can have a much thinner drift region. The thinner and more heavily doped n-pillar significantly reduces the *R*_SP_ of the SJ-MOSFET.

**Figure 4:**
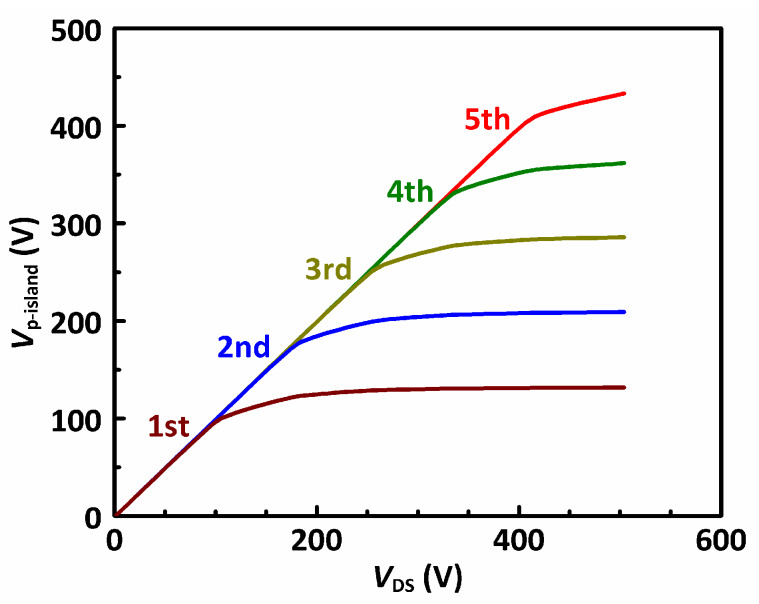
The voltage of the 1st to 5th p-island from top to bottom in the proposed MOSFET vs. *V*_DS_ in off-state.

In the proposed MOSFET, the n-drift region is heavily doped for a reduced *R*_SP_, which leads to a quick rise of the electric field peak. To obtain a higher *BV*, the proposed MOSFET uses multiple p-islands at the sidewall of the trench. The equal-potential lines of the proposed MOSFET, shown in Figure 2c, are crowded at not only the p-body/n-drift junction but also every p-island/n-drift junction. Hence, the electric field in the proposed MOSFET has multiple peaks. The proposed MOSFET was designed by first assuming that there is no p-island. When a drain voltage *V*_DS_ is applied, a depletion region is formed in the drift region until breakdown occurs at *V*_DS_ = *BV*_0_. At *BV*_0_, if the width of the depletion region is *W*_0_, the distance between the p-body and the 1st p-island is slightly smaller than *W*_0_ to allow some design margin. Therefore, the 1st p-island starts to support the off-state voltage before the p-body/n junction sees the critical breakdown field. Following the same manner, the second p-island, third p-island, etc. are designed until the required blocking voltage is obtained. In the design of the p-islands, the p-island should be located within the previous p-island’s depletion region by a certain margin to allow some process variation. If the p-island is placed out of the previous p-island’s depletion region, the electric field around the previous p-island will reach the critical breakdown field before the next p-island starts to clamp the voltage. These voltage clamping effects of the p-islands are similar to those in the floating field rings widely adopted for the edge termination of power devices [27,28]. Figure 4 shows the potential of the p-islands in the proposed MOSFET as a function of the off-state drain voltage.

**Figure 5:**
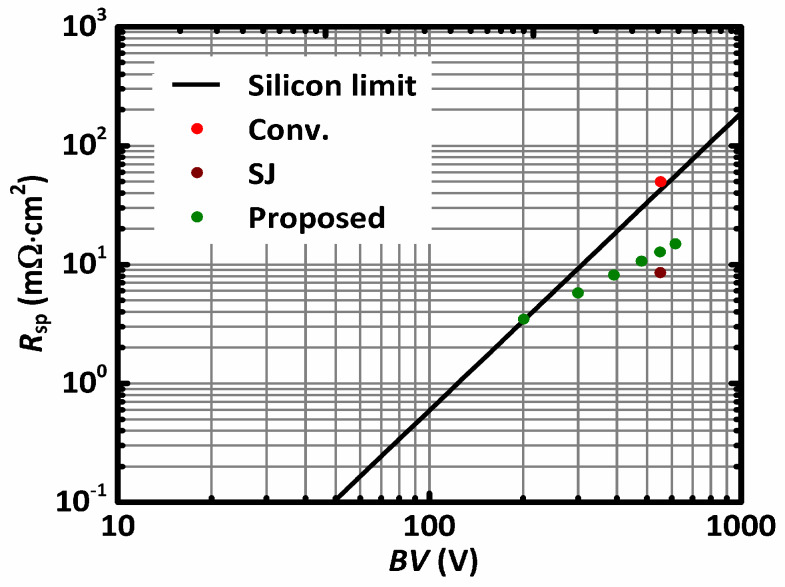
Relationship between *R*_SP_ and *BV* of the MOSFETs and the 1D “silicon limit.”

Unlike traditional MOSFET, which reduces the doping concentration when a higher *BV* is required, the proposed MOSFET structure uses a high doping concentration even for increased *BV*. According to the above analysis, to increase the *BV* of the proposed MOSFET, the doping concentration of the n-drift region can be kept unchanged, and the thickness of the n-drift region roughly scales with *BV*. The proposed MOSFET roughly follows *R*_SP_ ∝ *BV* as contrasted by *R*_SP_ ∝ *BV*^2.5^ for the conventional power MOSFET. Thus, the *R*_SP_ of the proposed MOSFET rises much slower as *BV* increases. Figure 5 benchmarks *R*_SP_ and *BV* for the conventional power MOSFET, the SJ-MOSFET, and the proposed MOSFET. The proposed MOSFETs with a different number of p-islands are benchmarked. Both the SJ-MOSFET and the proposed MOSFET achieve a *R*_SP_-*BV* trade-off beyond the so-called 1D-limit. In this paper, the MOSFETs with *BV* = ~550 V were chosen for detailed study.

**Figure 6:**
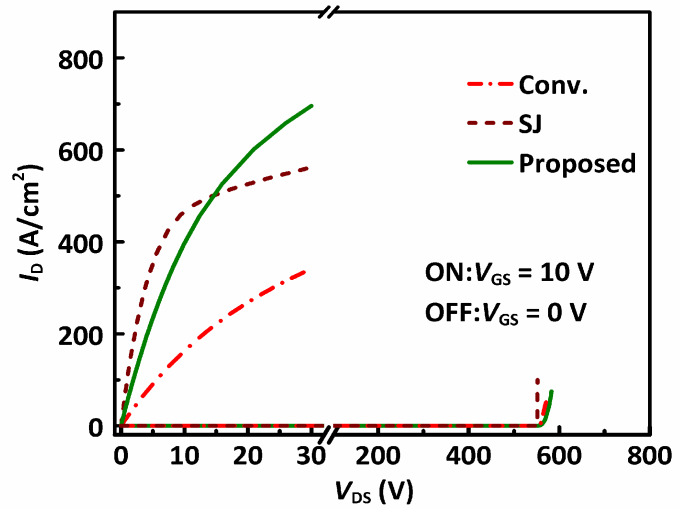
*I-V* characteristics of the studied MOSFETs.

The output characteristics of the studied MOSFETs are plotted in Figure 6. All the three MOSFETs present the same *BV* of ~550 V. In on-state with *V*_GS_ = 10 V, and the conventional MOSFET has a high *R*_SP_ of 49.80 mΩ·cm^2^. The SJ-MOSFET has a small *R*_SP_ of 8.52 mΩ·cm^2^ owing to the high doping concentration and short pillars compared to the n-drift region of the conventional power MOSFET. The proposed MOSFET features the same high doping concentration for the n-drift compared to the n-pillar in the SJ-MOSFET. The thickness of the n-drift region of the proposed MOSFET is the same as that of the conventional MOSFET, but thicker compared to the n-pillar in SJ-MOSFET. Thus, the proposed MOSFET has a much lower *R*_SP_ (12.74 mΩ·cm^2^) than the conventional MOSFET, although slightly higher than the SJ-MOSFET.

## 3. Body Diode Characteristics

**Figure 7:**
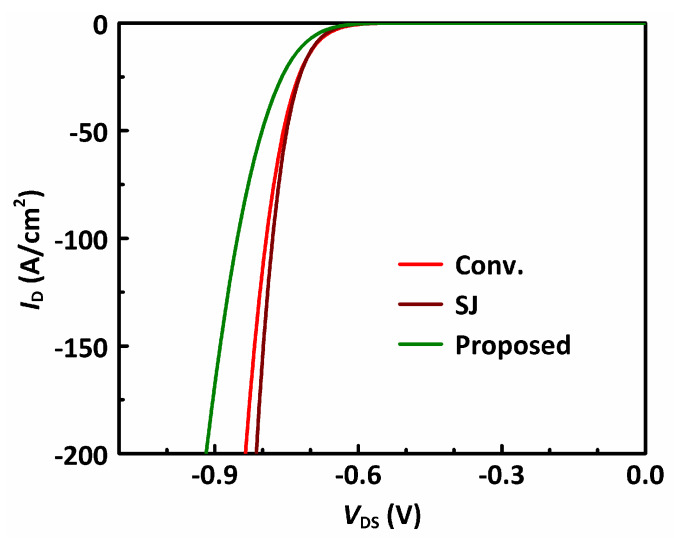
The reverse conduction characteristics of the studied MOSFETs.

Figure 7 shows the reverse conduction characteristics of the studied MOSFETs under *V*_GS_ = 0 V. The reverse conduction takes place through the internal parasitic PN junction. The reverse conduction voltage (*V*_RC_ = |*V*_DS_| at *I*_D_ = −100 A/cm^2^) of the conventional power MOSFET is 0.79 V, and that for the SJ-MOSFET is 0.78 V. The *V*_RC_ of the proposed MOSFET (0.85 V) is slightly higher than the other two MOSFETs since a portion of its device area is taken by the trench, which does not contribute to the reverse conduction. In a practical application with an optimized dead time design, the influence of this slightly higher *V*_RC_ is limited.

**Figure 8:**
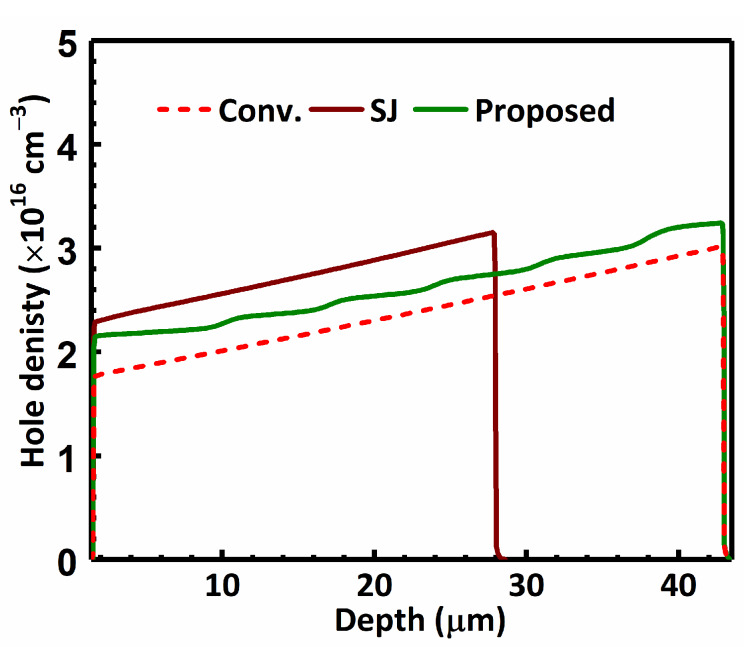
Hole density along the depth of the studied MOSFETs at *I*_D_ = −100 A/cm^2^.

Figure 8 plots the minority carrier density along the depth of the studied MOSFETs at the reverse conduction current of 100 A/cm^2^. All the studied MOSFETs have a similar level of minority carrier density.

In modern power switching applications, the reverse recovery performance of the power MOSFETs is of crucial importance [13,29,30,31]. During reverse conduction, a large amount of minority carriers is injected into the drift region of the MOSFETs. Before the power MOSFETs are switched to sustain the forward off-state voltage, the polarity of the voltage across the device is reversed, and the devices experience the reverse recovery process. During this process, the minority carriers have to be removed from the drift region so that the device can be able to block the forward off-state voltage.

**Figure 9:**
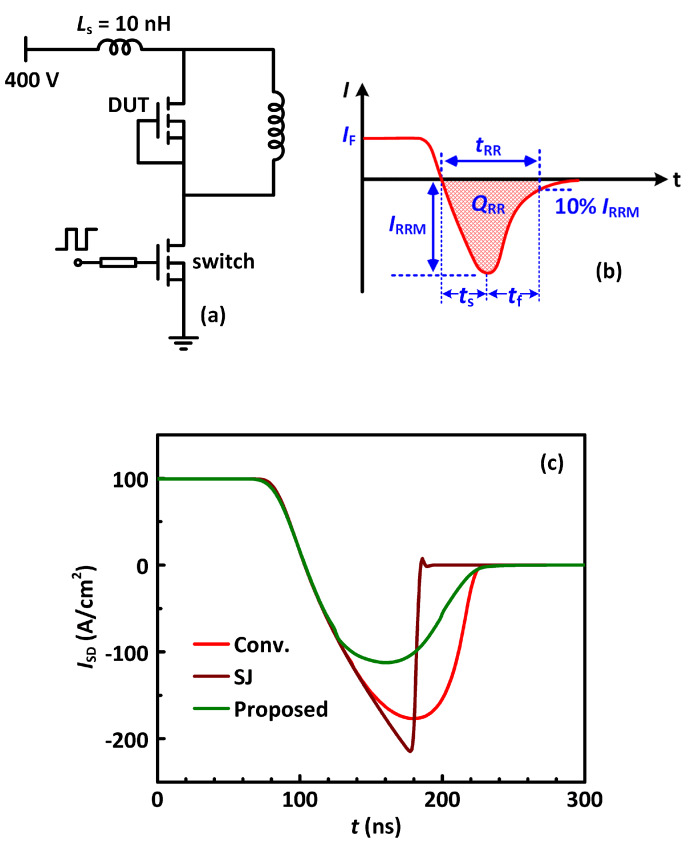
(**a**) Test circuit configuration for the reverse recovery characteristics. (**b**) Schematic of reverse recovery waveforms. (**c**) Reverse recovery characteristics of the studied MOSFETs.

The reverse recovery performance of the MOSFETs was studied using the testing circuit in Figure 9a. The supply voltage is 400 V, and a load current of 100 A is adopted. The device-under-test (DUT) is at the high-side of the circuit. A conventional MOSFET is employed as the switch in the circuit for all reverse recovery tests. Both the DUT and the switch have an area of 1 cm^2^.

Figure 9b illustrates a typical reverse recovery behavior of a power MOSFET. The reverse recovery charge (*Q*_RR_) is the integration of the reverse recovery current. The reverse recovery in the DUT causes an appreciable switching loss not in the DUT itself, but in the low-side power transistor. The reverse recovery current of the DUT adds up with the load current during the switching transient, leading to an increase of the switching current in the low-side transistor, which consequently causes an increase of power loss. The reverse recovery charge (*Q*_RR_) of the DUT is an integration of its reverse recovery current over the switching time duration and is a critical parameter to evaluate the power MOSFET [13]. The softness of the reverse recovery process is defined by *S* = *t*_f_/*t*_s_, where the *t*_f_ and *t*_s_ are illustrated in Figure 9b. The softness of the reverse recovery process is also of crucial importance. A snappy reverse recovery (i.e., a reverse recovery with very small *S*, marked by a sharp falling of current after the peak reverse recovery current) causes a very sharp change of current in the power loop of the circuit, which is very like to induce voltage spikes and oscillations due to the existence of parasitic inductances in the circuit. Therefore, a relatively softer reverse recovery (with larger *S*) is usually desired [13,32].

Figure 9c plots the reverse recovery performances of the studied MOSFETs. The reverse recovery charge *Q*_RR_ of the conventional power MOSFET is 14.3 µC/cm^2^, with a softness of 0.57. The SJ-MOSFET has a smaller *Q*_RR_ of 9.85 µC/cm^2^ owing to the thinner drift region. However, the softness of the SJ-MOSFET is as low as 0.07. Such a snappy reverse recovery is caused by the large aspect PN junction area in the SJ-MOSFET; the large-area PN junction extracts the minority carriers through the whole depth of the drift region. When all the minority carriers are quickly removed, the reverse recovery current falls abruptly. The snappy reverse recovery is deleterious for the system reliability and prevents the SJ-MOSFET technology to be adopted in many power switching applications. The proposed MOSFET has a *Q*_RR_ of 8.88 µC/cm^2^. *Q*_RR_ of the proposed MOSFET is smaller than the conventional power MOSFET, although they have a similar level of minority carrier density because a portion of the active area of the proposed MOSFET is consumed by the trench that does not store minority carriers. The softness of the proposed MOSFET is 0.90, larger than both the SJ-MOSFET and the conventional power MOSFET.

The main performances of the studied MOSFETs are listed in Table 2 for a comparison. Both the SJ-MOSFET and the proposed MOSFET can obtain a beyond-1D-limit *R*_SP_-*BV* trade-off. However, the superior reverse recovery performance of the proposed MOSFET makes it a preferable candidate for power switching applications where hard commutation is required [30,33].

## 4. Proposed Process Flow

**Figure 10:**
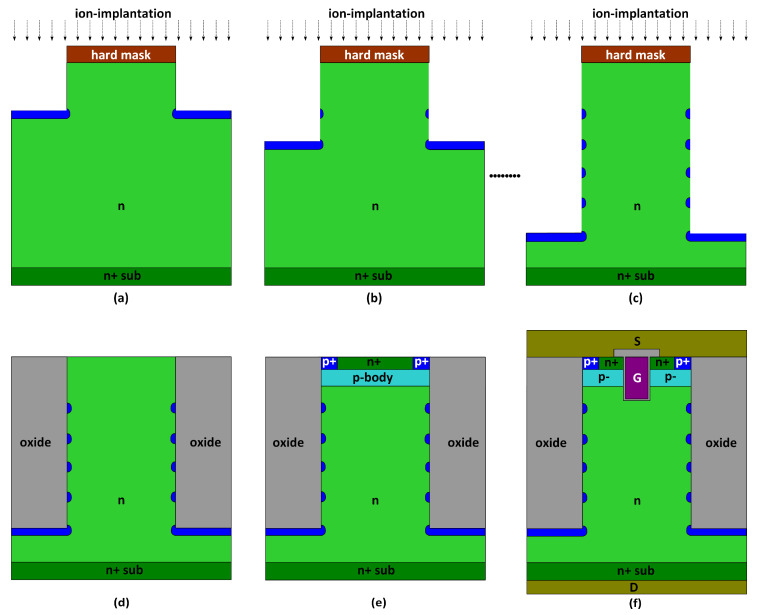
Proposed process flow for the MOSFET featuring trench oxide and p-islands: (**a**) patterned silicon oxide and the topmost p-island formation by etching and ion-implantation; (**b**) second topmost p-island definition in the same manner; (**c**) the other p-islands formation by repeated etching and ion-implantation, and a thermal process to activate the dopants and further enhance the lateral spread of the dopants; (**d**) trench oxide growth and etching back; (**e**) p-body, p+-source, n+-source as well as (**f**) trench gate and contact formation in the same manner as the conventional trench MOSFET.

The proposed MOSFET can be realized using a similar approach as a conventional power MOSFET. The key difference between the proposed MOSFET and the conventional power MOSFET is the trench and the p-islands. Figure 10 presents a proposed fabrication approach for the new device structure. In Figure 10a, with a patterned hard mask (e.g., silicon oxide), the epitaxial n-drift layer is dry-etched for a designed depth. The deep trench can be formed using the deep reactive ion etching (DRIE) technology, which is widely used in MEMS, memory, and power devices. The DRIE process consists of multiple cycles of dry etching and passivation. The passivation layer suppresses the lateral etching of the silicon material and helps to form fine vertical trenches [34,35,36,37,38]. A p-type ion implantation is performed, which creates a p-type region under the trench with a certain lateral spread. Then, in Figure 10b, the above etch and implantation processes are repeated to form the second p-island. In Figure 10c, the above processes are repeated multiple times to obtain the targeted number of p-islands. At last, thermal annealing will be performed to further enhance the lateral spread of the dopants. In Figure 10d, the trench is filled with oxide, and the surface is planarized. In Figure 10e, the surface implantations are carried out to form the p-body and n+ source region. Finally, in Figure 10f, the gate structure and metallization are implemented. According to the proposed process, the new structure can be realized using mature silicon fabrication technology.

## 5. Conclusions

The performance of the conventional power MOSFET is limited by the *R*_SP_-*BV* trade-off relationship: *R*_SP_ ∝ *BV*^2.5^, which is also known as the 1D-silicon-limit. The SJ-MOSFET breaks the 1D-limit but suffers a snappy reverse recovery that limits its application in many applications, where hard commutation is required. In this work, a new power MOSFET architecture is proposed, which simultaneously offers a beyond-1D-limit *R*_SP_-*BV* trade-off and a superior reverse recovery performance. The proposed MOSFET has a much higher doping concentration compared to the conventional power MOSFET, which reduces the *R*_SP_. A high breakdown voltage is obtained in the proposed MOSFET by splitting the single electric field peak into multiple peaks using multiple p-islands along a deep trench. The snappy reverse recovery inherent to SJ-MOSFET is avoided in the newly proposed MOSFET. Therefore, the proposed MOSFET can be used in various power switching applications where hard commutation is required.

## Figures and Tables

**Table 1:** Key parameters of the studied MOSFETs.

	Parameters	Value	Unit
Conventional MOSFET	doping of n-drift	3.8 × 10^14^	cm^−3^
	thickness of n-drift	42	µm
	cell pitch	3	µm
Superjunction MOSFET	doping of n/p-pillar	4 × 10^15^	cm^−3^
	thickness of n/p-pillar	28	µm
	pitch of n/p-pillar	3	µm
Proposed MOSFET	doping of n-drift	4 × 10^15^	cm^−3^
	thickness of n-drift	42	µm
	pitch of n-drift	3	µm
	pitch of trench oxide	3	µm

**Table 2:** Comparison of the device characteristics.

	Conv.	SJ.	Proposed	Unit
*R*_SP_	49.80	8.52	12.74	mΩ·cm^2^
*BV*	550	550	550	V
*V*_RC_^a^	0.79	0.78	0.85	V
*t*_RR_	116	80	114	ns
*t*_s_	74	75	60	ns
*t*_f_	42	5	54	ns
*S* (*t*_f_/*t*_s_)	0.57	0.07	0.90	-
*Q*_RR_	14.30	9.85	8.88	µC/cm^2^
*I*_RRM_	177	216	114	A/cm^2^

^a^*V*_RC_ = |*V*_DS_| at *I*_D_ = −100 A/cm^2^.
